# Extracellular fluid, cerebrospinal fluid and plasma biomarkers of axonal and neuronal injury following intracerebral hemorrhage

**DOI:** 10.1038/s41598-021-96364-x

**Published:** 2021-08-20

**Authors:** Lovisa Tobieson, Henrik Zetterberg, Kaj Blennow, Niklas Marklund

**Affiliations:** 1 Department of Neurosurgery in Linköping, and Department of Biomedical and Clinical Sciences, Linköping University, Linköping, Sweden; 2 Department of Psychiatry and Neurochemistry, Institute of Neuroscience and Physiology, The Sahlgrenska Academy, University of Gothenburg, Mölndal, Sweden; 3 Clinical Neurochemistry Laboratory, Sahlgrenska University Hospital, Mölndal, Sweden; 4 Department of Neurodegenerative Disease, UCL Institute of Neurology, London, UK; 5 UK Dementia Research Institute at UCL, London, UK; 6 Department of Clinical Sciences Lund, Neurosurgery, Skåne University Hospital, Lund University, Lund, Sweden

**Keywords:** Stroke, White matter injury, Neurodegeneration, Stroke, Mechanisms of disease, Stroke, Cell death in the nervous system, Blood-brain barrier

## Abstract

Spontaneous intracerebral hemorrhage (ICH) is the most devastating form of stroke. To refine treatments, improved understanding of the secondary injury processes is needed. We compared energy metabolic, amyloid and neuroaxonal injury biomarkers in extracellular fluid (ECF) from the perihemorrhagic zone (PHZ) and non-injured (NCX) brain tissue, cerebrospinal fluid (CSF) and plasma. Patients (n = 11; age 61 ± 10 years) undergoing ICH surgery received two microdialysis (MD) catheters, one in PHZ, and one in NCX. ECF was analysed at three time intervals within the first 60 h post- surgery, as were CSF and plasma samples. Amyloid-beta (Aβ) 40 and 42, microtubule associated protein tau (tau), and neurofilament-light (NF-L) were analysed using Single molecule array (Simoa) technology. Median biomarker concentrations were lowest in plasma, higher in ECF and highest in CSF. Biomarker levels varied over time, with different dynamics in the three fluid compartments. In the PHZ, ECF levels of Aβ40 were lower, and tau higher when compared to the NCX. Altered levels of Aβ peptides, NF-L and tau may reflect brain tissue injury following ICH surgery. However, the dynamics of biomarker levels in the different fluid compartments should be considered in the study of pathophysiology or biomarkers in ICH patients.

## Introduction

Spontaneous intracerebral hemorrhage (ICH) is a severe form of stroke with < 40% of patients returning to independently caring for activities of daily living^1^. The ICH causes a primary injury to brain tissue caused by shearing forces and pressure from the extravasated blood along with a possible acute rise in intracranial pressure, compromising cerebral blood flow. The brain tissue surrounding the ICH, the perihemorrhagic zone (PHZ), may be particularly vulnerable to secondary injury mechanisms triggered by the blood breakdown products. The PHZ is characterised by hypoperfusion and metabolic disturbances indicative of mitochondrial dysfunction^2^ and an inflammatory response^3^.

To monitor the secondary injury processes, cerebral microdialysis (MD) has long been used in neurocritical care, measuring energy-metabolic markers such as glucose, lactate, pyruvate, glutamate and glycerol in extracellular fluid (ECF). With the introduction of large pore MD catheters, it became possible to also sample and monitor macromolecules such as cytokines^4,5^, proteins^6^, and biomarkers of neuronal and axonal injury. Biomarkers in ECF such as amyloid-beta peptides (Aβ) and tau have been sampled by MD in patients suffering from subarachnoid hemorrhage (SAH)^7–10^ and traumatic brain injury (TBI)^9,11–16^, although no previous studies exist in ICH patients^16^. Furthermore, neurofilament light (NF-L) is emerging as a promising biomarker of large calibre axonal injury in serum or cerebrospinal fluid (CSF)^17–20^ and occasionally also by MD^9^ in patients with a variety of neurological disorders.

The primary and secondary injury processes in ICH result in both neuronal and white matter injury^21^ which may be progressive and insights derived from brain tissue monitored by MD may be useful in the development of novel therapies. Since MD is an invasive technique, it would be preferable if the dynamics of secondary injury processes could be monitored in CSF or blood samples. There are only very few studies^13^ comparing the levels of brain injury biomarkers in different bodily fluids of patients. In the present study, we aimed to compare levels of biomarkers in three compartments; ECF, CSF and plasma at three time points during the initial 60 h following ICH surgery. We also hypothesised that the levels of Aβ, tau and NF-L, monitored by MD, would be different in the vicinity of the ICH (the perihemorrhagic zone; PHZ), when compared to those in the non-injured cortex (NCX).

## Methods

### Ethics

The regional ethical committee in Linköping, Sweden approved the study protocol (decision number 2014/236-31), and the study was carried out in accordance with relevant guidelines and regulations, including the World Medical Association (WMA) Declaration of Helsinki. Since the patients could not themselves consent to the study, a written informed consent was obtained from the patient's closest relative.

### Patients

Adult patients admitted to the Neurosurgical Department of University Hospital of Linköping, Sweden, requiring emergent surgery with craniotomy for spontaneous intracerebral hemorrhage (ICH) were prospectively included in the study^2^. The ICH was surgically evacuation by routine microneurosurgical technique^2^ and patients received an external ventricular drain (EVD; DePuy Synthes, Raynham, USA) for monitoring intracranial pressure. One microdialysis (MD) catheter (CMA-71 Brain Catheter, M-Dialysis, Solna, Sweden) was inserted, at a 45-degree angle, via the craniotomy into the perihemorrhagic zone (PHZ) defined as < 1 cm of the evacuated ICH, and one catheter was inserted in non-eloquent and non-injured cortex (NCX) remote to the ICH; either ipsilateral to the ICH or in the contralateral hemisphere via a separate frontal burr hole. MD catheter locations were verified with a post-operative CT-scan.

Seven of the patients were included in a previous publication from our group evaluating energy metabolic disturbances following ICH surgery^2^.

Pre- and post-operatively the patients were treated by a standardized neurocritical care protocol as previously described^2^. None of the patients had any known neurodegenerative disorder, dementia or Alzheimer’s disease at time of ICH onset.

### Plasma and CSF sampling

Blood plasma was drawn daily through an arterial line catheter. CSF samples were drawn from the EVD daily following discarding of the initial 2 mL of CSF. Plasma and CSF samples were then centrifuged at 1800 G for 10 min at 4 degrees Celsius and the supernatant was decanted into 1 mL aliquots and stored at -80 degrees C until further analysis.

### Microdialysis

Microdialysis catheters of 10 mm length with a molecular weight cut-off of 100 kDa (CMA-71, M-dialysis AB, Solna, Sweden) were used in accordance with department routines. Catheters were perfused with 5% human albumin in a solution containing the excipients sodium chloride, N-acetyl-DL-tryptophan and caprylic acid (Albunorm, 50 g/l, Octapharma AB, Stockholm, Sweden), at a rate of 0.3 µL/min using the CMA 106 perfusion pump (M-Dialysis AB, Solna, Sweden)^4^. The first 2 h of sampling were discarded according to consensus praxis^22,23^. Microdialysis samples were collected every 2 h for routine analysis of small molecular metabolites (glucose, lactate, pyruvate, glycerol and glutamate)^22–25^. Following this analysis, the remaining MD sample (approximately 30 µL/vial) was frozen and stored at − 20 °C, and typically within 2–8 weeks transferred to Eppendorf vials and stored at − 86 °C until further analysis.

Plasma, CSF and microdialysis samples were analysed at median (range) 14 (4–22) hours, 38 (28–42), and 59 (52–68) hours after surgery.

### Analytical methods

#### Analysis of energy-metabolic markers

The ISCUS Flex® analyser (M Dialysis AB, Solna, Sweden) was used bedside in the neurointensive care unit (NICU) to determine extracellular levels of glucose, lactate, pyruvate, glycerol and glutamate immediately after sample collection. As per the manufacturer’s instructions the lower limit of detection (LLOD) was 1.0 μmol/L for glutamate, 0.1 mmol/L for glucose and lactate, 10 μmol/L for pyruvate and 0.22 mg/mL for glycerol. Metabolite concentrations in the MD samples were analysed by the enzymatic method. Sample volume required was 0.5 µL for glucose, 0.2 µL for lactate, 0.5 µL for pyruvate, 0.5 µL for glycerol, 1 µL for glutamate and 0.5 µL for urea, leaving approximately 30 µL for the biomarker analysis.

#### Analysis of neuroaxonal injury biomarkers

All fluid samples were analysed for Aβ40, Aβ42, tau and NfL using commercially available assays on the Single molecule array (Simoa) HD-1 Analyser (Quanterix, Billerica, MA). Specifically, NfL concentration was measured using the NF-Light kit, whilst Aβ40, Aβ42, and tau concentrations were measured using the Neurology 3-Plex panel (Quanterix Billerica, MA). Plasma samples were diluted fourfold, according to the kit inserts. CSF and microdialysis samples were diluted 200- or 400-fold (depending on analyte concentration). All measurements were performed in one round of experiments by board-certified laboratory technicians who were blinded to clinical data. Intra-assay coefficients of variation were below 10%.

### Statistical methods

As biomarker levels were non-normally distributed, non-parametric Kruskal–Wallis test was employed for multiple group comparisons, and Friedman’s test for repeated measures when biomarker levels were compared over time. To correct for multiple comparisons the Bonferroni method was used. Wilcoxon signed rank test was employed to compare two related groups. Correlations were investigated using Spearman’s rho (ρ). All statistical tests were 2-sided and the significance threshold was set at *p* < 0.05. Normally distributed data are presented as mean and standard deviation (SD). Non-normally distributed data are presented as median and range or median and interquartile range (IQR). Statistical analysis was performed using IBM SPSS 27.0 (IBM, Kista, Sweden).

## Results

Eleven patients (3 females and 8 males) were included. Mean age was 61.4 (± 10) years. Seven patients had a deep intracerebral haemorrhage originating from the basal ganglia or external capsule, whereas four patients had a lobar haemorrhage. Mean ICH volume was 73.5 (± 13) mL. Median time from ICH onset to surgery was 10 (6–74) hours and the mean microdialysis (MD) sampling time was 90.4 (± 36) hours. The mean distance from MD catheter to the evacuated ICH was 6.3 (± 2) mm for the perihemorrhagic zone (PHZ) catheter and 20.8 (± 10) mm for the non-injured cortex (NCX) catheter. Three patients had the NCX catheter placed in the hemisphere contralateral to the ICH (Table 1). None of the patients included in the study had dementia or Alzheimer’s disease. No patient had a prior or post-ICH onset a suspicion of cerebral amyloid angiopathy (CAA)^26^.

**Table 1:** Patient characteristics.

Patient #	Age (years)	Sex (M/F)	ICH size (mL)	Time to surgery (hours)	Duration MD-sampling (hours)	Distance to ICH PHZ-MD (mm)	Distance to ICH NCX-MD (mm)
1	48	M	57	8	172	5	13
2	55	M	87	12	98	7	13
3	68	M	90	10	108	3	34
4	68	F	77	10	104	7	19
5	53	F	66	6	84	6	17
6	67	M	66	22	42	4	15
7	43	M	88	8	132	9	33*
8	66	F	88	40	56	10	72*
9	65	M	74	38	80	9	15
10	64	M	59	8	54	4	40
11	78	M	56	74	64	5	75*

*M* male, *F* female, *ICH* intracerebral haemorrhage, *MD* microdialysis, *PHZ* perihemorrhagic zone, *NCX* non-injured cortex.

*Contralateral to ICH.

### Energy-metabolic markers

**Figure 1:**
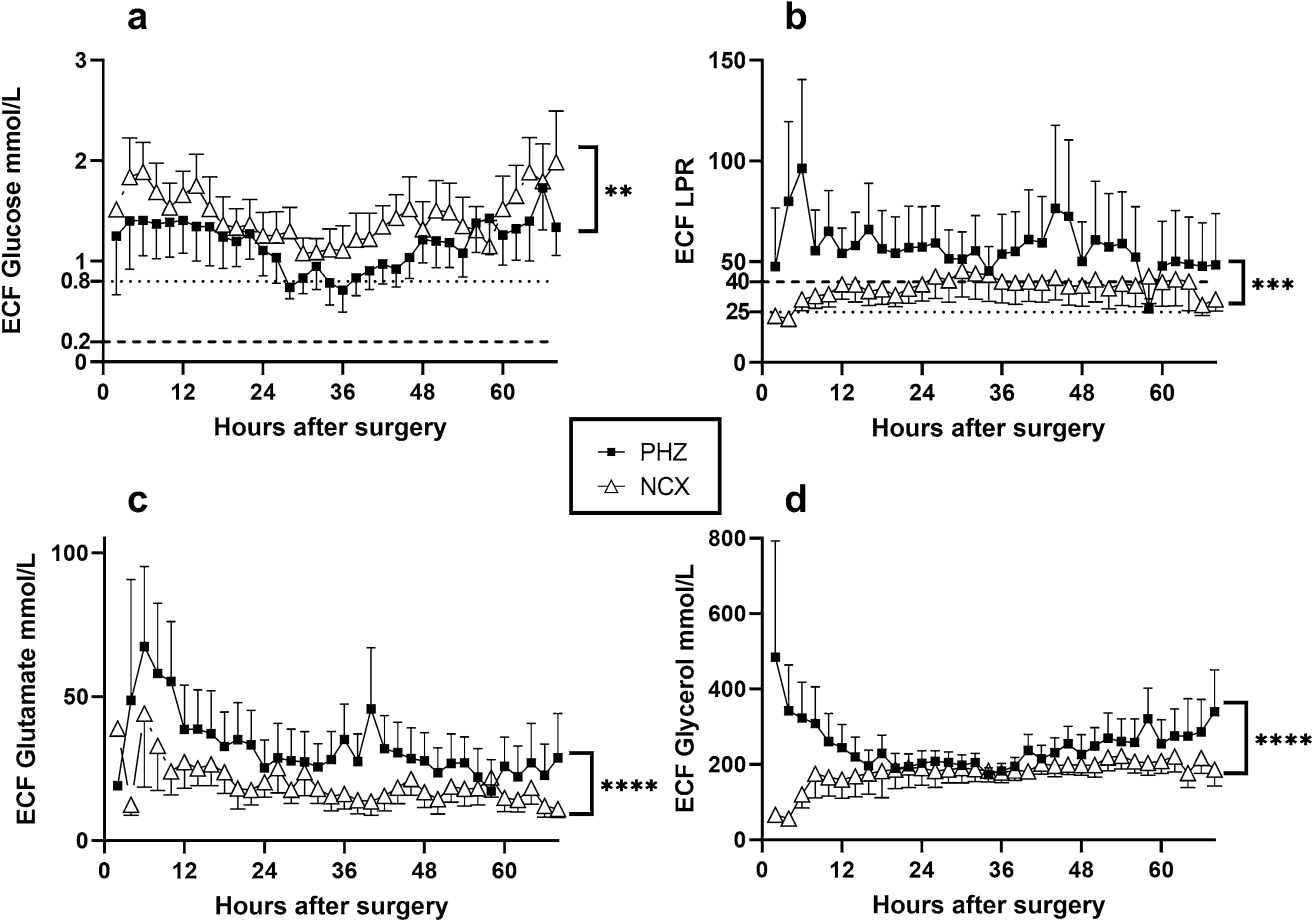
Energy-metabolic markers in extracellular fluid. (**a**) Extracellular fluid (ECF) level of glucose was significantly lower in the perihemorrhagic zone (PHZ) compared to non-injured cortex (NCX) during the initial 68 h of monitoring (*p* = 0.004), although both levels were above critical level in both locations^23^. (**b**) Lactate pyruvate ratio (LPR) was significantly higher in the PHZ compared to the NCX (*p* = 0.001) as were the (**c**) extracellular levels of glutamate and (**d**) glycerol (*p* < 0.0001). In addition, ECF levels of lactate and pyruvate were significantly higher in PHZ compared to NCX (*p* < 0.0001; data not shown). Mean and S.E.M. presented for clarity. ** = *p* ≤ 0.01, *** = *p* ≤ 0.001, **** = *p* < 0.0001. ECF = extracellular fluid; LPR = lactate pyruvate ratio; PHZ = perihaemorrhagic zone; NCX = non-injured cortex.

The initial 68 h of monitoring of the energy metabolites revealed significantly lower extracellular fluid (ECF) level of glucose in the PHZ compared to the NCX (Fig. 1a; *p* = 0.004), however, levels were above critical in both locations^23^. ECF level of lactate was significantly higher in the PHZ when compared to the NCX (data not shown; *p* < 0.0001), as was the pyruvate (data not shown; *p* < 0.0001), lactate/pyruvate ratio (LPR; Fig. 1b; *p* = 0.001), glycerol (Fig. 1c; *p* < 0.0001) and glutamate (Fig. 1d; *p* < 0.0001).

### Differences in extracellular levels of Aβ, tau and NF-L between PHZ and NCX

**Figure 2:**
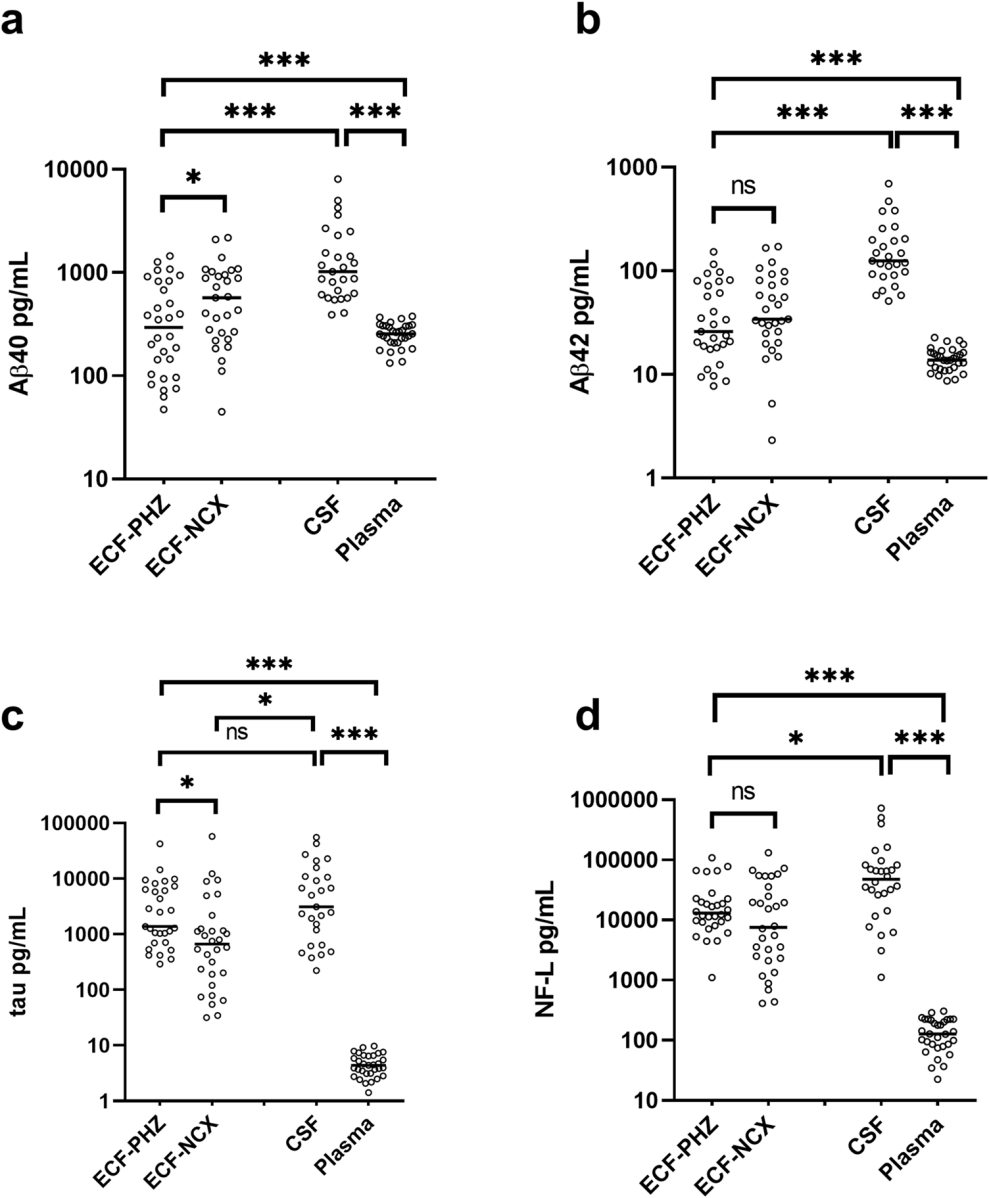
Aβ40 levels were lower, and tau levels higher, in perihemorrhagic zone (PHZ) when compared to non-injured cortex (NCX). All biomarker levels were lower in plasma, and higher in cerebrospinal fluid (CSF), compared to the extracellular fluid (ECF). (**a**) There were significantly lower levels of Aβ40 in extracellular fluid (ECF) of perihemorrhagic tissue (ECF-PHZ) compared to non-injured cortex (ECF-NCX; *p* = 0.048). Levels of Aβ40 were higher in CSF than ECF and plasma. (**b**) ECF levels of Aβ42 did not differ significantly between the PHZ and NCX. CSF levels of Aβ42 were higher than ECF and plasma levels. (**c**) ECF levels of tau were significantly higher in PHZ compared to NCX (*p* = 0.031). CSF levels of tau were higher than ECF from NCX but not PHZ (*p* = 0.209), and were higher than plasma levels of tau. (**d**) There was no significant difference in levels of Neurofilament light (NF-L) in ECF from PHZ compared to NCX. CSF levels of NF-L were significantly higher than in ECF and plasma. Aβ = amyloid-beta; Tau = microtubule-associated protein tau; NF-L = neurofilament light; ECF-PHZ = extracellular fluid of perihemorrhagic zone; ECF-NCX = extracellular fluid of non-injured cortex; CSF = cerebrospinal fluid; ECF = extracellular fluid; ns = not significant. * = *p* < 0.05; *** = *p* < 0.001.

The extracellular fluid (ECF) concentration of Aβ40 was lower in the PHZ compared to NCX (*p* = 0.048; Fig. 2a), whereas there was no significant difference in the ECF concentrations of Aβ42 (*p* = 0.065; Fig. 2b). The concentration of tau was higher in the PHZ compared to NCX (*p* = 0.031; Fig. 2c), while there was no significant difference in neurofilament-light (NF-L; *p* = 0.199; Fig. 2d).

### Aβ, tau and NF-L levels in the different bodily fluid compartments

Median [IQR] concentrations of Aβ40 were 385.0 [684] pg/mL in microdialysate from the perihaemorrhagic zone (PHZ), 727.0 [742] pg/mL in microdialysate from non-injured cortex (NCX), 255 [96] pg/mL in plasma and 875.0 [940] pg/mL in cerebrospinal fluid (CSF). Median [IQR] concentration of Aβ42 was 33.8 [61] pg/mL in PHZ, 47.2 [62] pg/mL in NCX, 13.8 [5] pg/mL in plasma, and 125.0 [113] pg/mL in CSF. Median [IQR] concentration of Tau was 2510.0 [7505] pg/mL in PHZ, 755.0 [1059] pg/mL in NCX, 4.4 [3] pg/mL in plasma and 6118.0 [14245] pg/mL in CSF. Median [IQR] concentration of NF-L was 11,868.0 [14633] pg/mL in PHZ, 7914.0 [33267] pg/mL in NCX, 128.0 [141] pg/mL in plasma and 64,469.0 [54573] pg/mL in CSF (Fig. 2a–d). Levels of all biomarkers were significantly higher in CSF and ECF than in plasma (*p* < 0.001; Fig. 2a–d).

### Aβ, tau and NF-L biomarker dynamics over time

**Figure 3:**
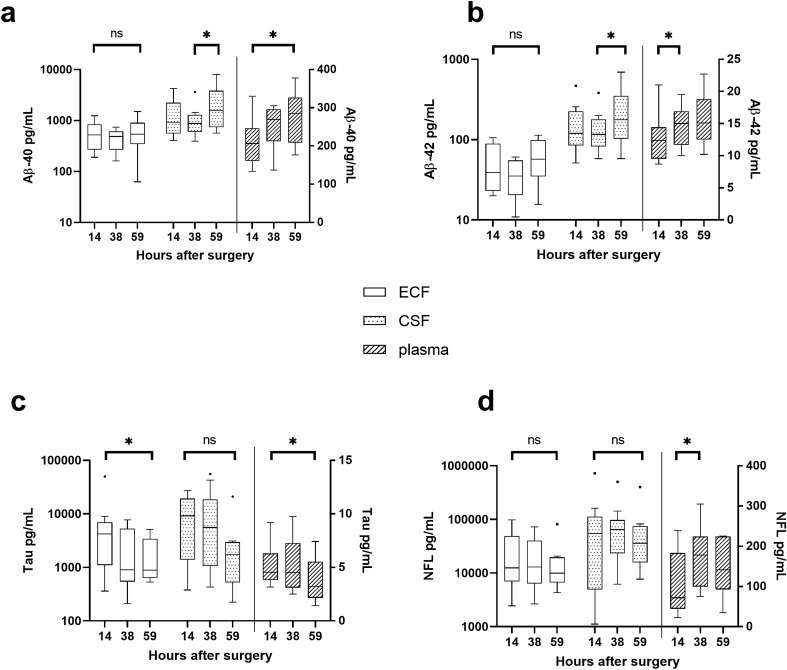
Dynamic changes in Aβ, tau and NF-L biomarkers in the different fluid compartments over time. In extracellular fluid (ECF), median levels of Aβ-40, Aβ42 or NF-L did not change over time, whereas the tau levels significantly decreased over time. In cerebrospinal fluid (CSF), Aβ40 and Aβ42 levels increased, whereas there was no significant difference in the levels of tau or NF-L over time. In plasma, levels of Aβ40 and Aβ42 increased over time, as did the levels of tau and NF-L. Tukey box plot. Note that, for visual clarity, ECF and CSF levels are displayed on the left Y-axis which is logarithmic whereas plasma levels are displayed on the right Y-axis which is linear. Ns = not significant; * = *p* < 0.05.

In the ECF, the median concentrations of Aβ40 and Aβ42 in PHZ and NCX did not change over time (*p* = 0.156 and *p* = 0.311, respectively; Fig. 3a,b). The median concentration of tau decreased between 14 (4–22) and 59 (52–68) hours after surgery (*p* = 0.023; Fig. 3c), whereas the median concentration of NF-L did not significantly change over time (*p* = 0.072; Fig. 3d).

Plasma concentration of Aβ40 increased between 14 (4–22) and 59 (52–68) hours after surgery (*p* = 0.029, Fig. 3a), and Aβ42 increased between 14 (4–22) and 38 (28–42) hours (*p* = 0.027, Fig. 3b). In contrast, plasma concentration of tau decreased between 14 (4–22) and 59 (52–68) hours after surgery (*p* = 0.045; Fig. 3c). Plasma concentration of NF-L increased between 14 (4–22) hours and 38 (28–42) hours (*p* = 0.029; Fig. 3d), but remained unchanged thereafter.

CSF concentrations of both Aβ40 and Aβ42 increased between 38 (28–42) hours and 59 (52–68) hours (*p* = 0.048 and 0.048 respectively; Fig. 3a,b). CSF concentrations of tau remained unchanged over time (*p* = 0.156; Fig. 3c), as did NF-L (*p* = 0.882; Fig. 3d).

### Correlations between biomarkers and energy metabolic markers in the extracellular fluid

**Figure 4:**
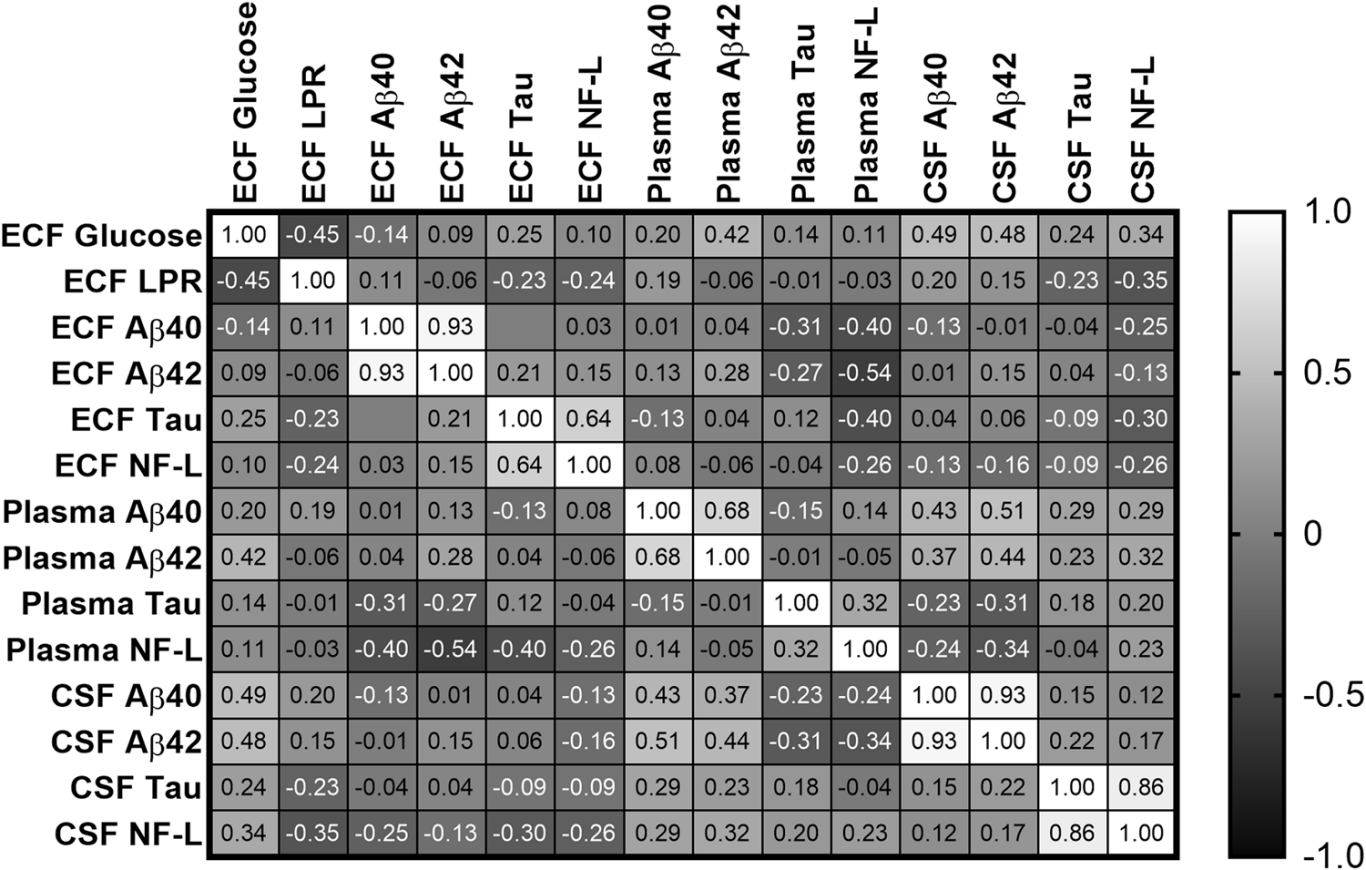
Correlations matrix between biomarkers in extracellular fluid (ECF), cerebrospinal fluid (CSF) and plasma. There was a strong correlation between levels of Aβ40 and Aβ -42 in ECF (ρ = 0.93, *p* < 0.05), plasma (ρ = 0.68, *p* < 0.05) and CSF (ρ = 0.93, *p* < 0.05), as for levels of tau and neurofilament light (NF-L) in ECF (ρ 0.64, *p* < 0.05) and CSF (ρ = 0.86, *p* < 0.05), whereas in plasma this correlation was moderate (ρ = 0.32, *p* < 0.05). ECF = extracellular fluid; LPR = lactate pyruvate ratio; Aβ = amyloid beta; NF-L = neurofilament light. Legend shows strong negative correlation in black and strong positive correlation in white.

Extracellular fluid (ECF) concentrations of Aβ40 and Aβ42 were highly correlated (ρ = 0.926, *p* < 0.001; Fig. 4), as were the concentration of tau and NF-L (ρ = 0.638, *p* < 0.001; Fig. 4). See Supplementary figure Ia for the linear regression of these correlations. There was no correlation, however, between ECF Aβ40 or Aβ42 and tau or between ECF Aβ40 or Aβ42 and NF-L. Levels of glucose in ECF were positively correlated with levels of Aβ40 and Aβ42 in CSF, respectively (ρ = 0.49 and ρ = 0.48, *p* = 0.01 for both; Fig. 4).

### Correlations between Aβ, NF-L and tau levels in plasma, CSF and ECF

Levels of Aβ-40, Aβ42, tau and NF-L in extracellular fluid (ECF) did not correlate with concentrations in neither plasma nor cerebrospinal fluid (CSF; Fig. 4).

Plasma levels of Aβ40 and Aβ42 showed a moderate correlation (ρ = 0.682, *p* < 0.001; Fig. 4; see Supplementary Figure Ib for linear regression of correlation), as opposed to plasma levels of tau and NF-L which did not correlate (*p* = 0.078). CSF levels of Aβ40 and Aβ42 showed a strong correlation (ρ = 0.926, *p* < 0.001; Fig. 4; see Supplementary Figure Ib for linear regression of correlation), as did tau and NF-L (ρ = 0.86, *p* < 0.001; Fig. 4; see Supplementary Figure Ib for linear regression of correlation).

Plasma levels and CSF levels of Aβ40 and Aβ42 showed a moderate correlation (ρ = 0.427, *p* = 0.026 and ρ = 0.443, *p* = 0.021, respectively; Fig. 4; see Supplementary Figure Ic for linear regression of correlation). Plasma and CSF levels of tau and NF-L were not correlated (*p* = 0.362 and *p* = 0.245, respectively).

## Discussion

In this study, the use of paired microdialysis (MD) enabled sampling of extracellular fluid (ECF) of the perihemorrhagic zone (PHZ) as well as of non-injured, control cortex (NCX) at a distance from the ICH. In addition, we also sampled cerebrospinal fluid (CSF) and plasma, and we measured markers of neuroaxonal injury in these three compartments over the first 60 h following surgery, which represents the time period when MD was clinically indicated as part of the multimodal monitoring in the neurocritical care setting which, for most ICH patients, is 2–4 days post-onset^27^. We observed that levels of Aβ40 were lower, and levels of tau higher, in the PHZ when compared to NCX. All biomarkers were measureable in the three compartments, with the highest levels observed in CSF, and lowest in plasma. Over time, the dynamics were different in the three compartments without any correlations across ECF, CSF and plasma, emphasising that for adequate interpretation of cerebral events leading to the release of a biomarker, knowledge of the relationship of biomarker levels in different compartments is crucial.

Similarly to previous results published by our group^2^, increased ECF lactate-pyruvate ratio (LPR) indicated a metabolic crisis in the PHZ. In addition, we found lower ECF Aβ-40, and higher ECF tau concentrations, in the PHZ without any correlations between biomarker levels and metabolic markers of metabolic distress. This could reflect PHZ pathophysiology, including edema formation, mitochondrial dysfunction, and inflammatory responses^28,29^. Similar to our findings of lower Aβ40 in the PHZ, a previous study of 18 traumatic brain injury (TBI) and SAH patients found decreased ECF levels of Aβ40 and Aβ42^10^ plausibly reflecting a decreased neuronal synaptic activity. In another TBI study, levels of Aβ40 and Aβ42 were higher in diffuse axonal injury (DAI) compared to focal TBI^15^. In these studies, the MD catheters were placed predominately in non-injured tissue, and not in brain tissue close to a focal lesion. Thus, these data are sampled from a less injured brain region than the PHZ region evaluated here.

We observed higher levels of the axonal injury biomarker tau in the PHZ compared to NCX, similar to findings of previous studies of TBI^9,14^, and SAH^7^ patients, whereas there was no difference in levels of NF-L in the PHZ compared to NCX.

Levels of Aβ-40, Aβ42, tau and NF-L were significantly higher in CSF compared to ECF and plasma, although all were measureable in all three compartments. Higher biomarker, in CSF than in ECF or plasma has also been found in previous studies in TBI and SAH patients^7,9,10^ possibly due to reduced relative recovery in ECF^10^. Following release of biomarkers produced by the brain injury into the ECF, monitoring by MD is preferable. However, the invasive nature, the small focal area that can be measured, and relatively poor time resolution of the method enables its use only in highly selected patients^30^. In TBI, biomarkers of neuroaxonal injury can be detected in ECF, CSF and plasma often in falling concentrations^31,32^. Our findings of lower levels of biomarkers in the ECF than in the CSF may reflect a reduced relative recovery across the MD membrane^30^, reduced recovery due to biomarker adsorption to surfaces^5,33^, physiological factors including tissue clearance^34^ and variability in blood–brain–barrier (BBB) integrity causing contamination by serum levels^35–37^. Peripheral plasma levels on the other hand, present the least invasive method although challenges include dilution effect of CNS derived biomarkers, and contamination by peripheral non-CNS production, reflected in our present study by significantly lower levels in plasma of all biomarkers.

ECF concentrations of Aβ did not fluctuate over time, implying a consistent production or release can a stable relative recovery be assumed^10^. We cannot exclude a decreased relative recovery masking any increased cerebral release of Aβ peptides, however^38^. In contrast, plasma and CSF levels of Aβ increased over the monitoring period, possibly reflecting an increased permeability of Aβ through the blood brain barrier (BBB)^39^, or increased peripheral production^40,41^.

Levels of tau decreased with time in ECF and plasma, and to some extent also in CSF, which could be reflective of a high level of axonal damage initially following ICH, which then decreases over the first 60 h following surgery.

Levels of neurofilament light (NF-L) were stable over time in ECF and CSF but showed significant increase in plasma over the initial monitored time period. Such an increase in plasma NF-L following acute brain injury has also been shown in several studies of a variety of neurological disorders^18,42–47^, with higher levels associated with more severe injury and poorer functional outcome.

There was a strong correlation between the levels of Aβ40 and Aβ42 within each compartment, which was expected since they share the same precursor protein (amyloid precursor protein; APP) and are typically secreted in a stable ratio^48^. Similarly, there was a correlation between ECF tau and NF-L—both considered markers of axonal injury- a finding in line with previous studies of SAH and TBI patients^9,49–52^. However, rather surprisingly, there was no correlation between individual levels of biomarkers in the ECF when compared to levels in CSF or plasma. This lack of correlation suggests caution when interpreting plasma or CSF biomarkers levels as indicators of ECF levels. Thus, more work is needed to understand the dynamics of evolving tissue using plasma or CSF biomarkers.

No previous study has to our knowledge compared levels and dynamics of Aβ, tau and NF-L in ECF, CSF and plasma^53^. In a previous study of six TBI patients, the levels of the F^2^-isoprostane 8-iso-prostaglandin F_2α,_ a biomarker of oxidative stress, were higher in ECF when compared to both plasma and ventricular CSF^13^, contrary to our present findings. A study of IL-6 levels in SAH patients showed higher levels in CSF than in ECF and lowest in plasma. Furthermore, IL-6 levels in CSF and ECF could predict neurologic deterioration which plasma levels could not^54^. As we have not explored relative recovery of each biomarker in this study, we cannot determine their true extracellular concentration. It is, however, plausible that the ECF concentration sampled by MD represents only a fraction of the true extracellular concentration which may be even higher than that observed in CSF. The sampling of Aβ from ECF is challenged by a tendency for Aβ to adsorb to microdialysis membrane, tubing, and vials which was avoided in our study by the use of Albumin in the perfusate^10^. Our set-up with paired catheters also allowed for a comparison of ECF biomarker levels between MD catheters, and presumably the relative recovery is similar between the NCX and PHZ.

## Conclusion

We found lower levels of extracellular Aβ40 and higher levels of extracellular tau in the perihemorrhagic zone (PHZ) when compared to non-injured cortex (NCX), during the first 60 h following surgical evacuation of intracerebral hemorrhage. These data suggest ongoing neuroaxonal injury in the PHZ, allowing for monitoring of the secondary injury process. Furthermore, median levels of Aβ40, Aβ42, tau and NF-L were higher in the cerebrospinal fluid than in ECF, which in turn was much higher than in plasma. We found poor correlation between levels of Aβ, tau and NF-L in the ECF when compared to CSF and plasma. Since the development of e.g. edema may be prolonged and monitoring with microdialysis not be feasible, studies of CSF and blood can be used. However, our results emphasize that for adequate interpretation of cerebral events leading to the release of a biomarker, knowledge of biomarker levels, dynamics and correlations in different compartments is crucial.

## Supplementary Information

Supplementary Information.

## Data Availability

The data supporting the findings in this study are available from the corresponding author, upon reasonable request.
