# Supplementary material for: Extracellular fluid, cerebrospinal fluid and plasma biomarkers of axonal and neuronal injury following intracerebral hemorrhage
Source: Sci Rep. 2021 Aug 20;11:16950. doi: 10.1038/s41598-021-96364-x (PMC8379247; doi:10.1038/s41598-021-96364-x)
Supplement: Supplementary file 1 — Supplementary Information. [file 41598_2021_96364_MOESM1_ESM.docx]

**Supplementary figure I**


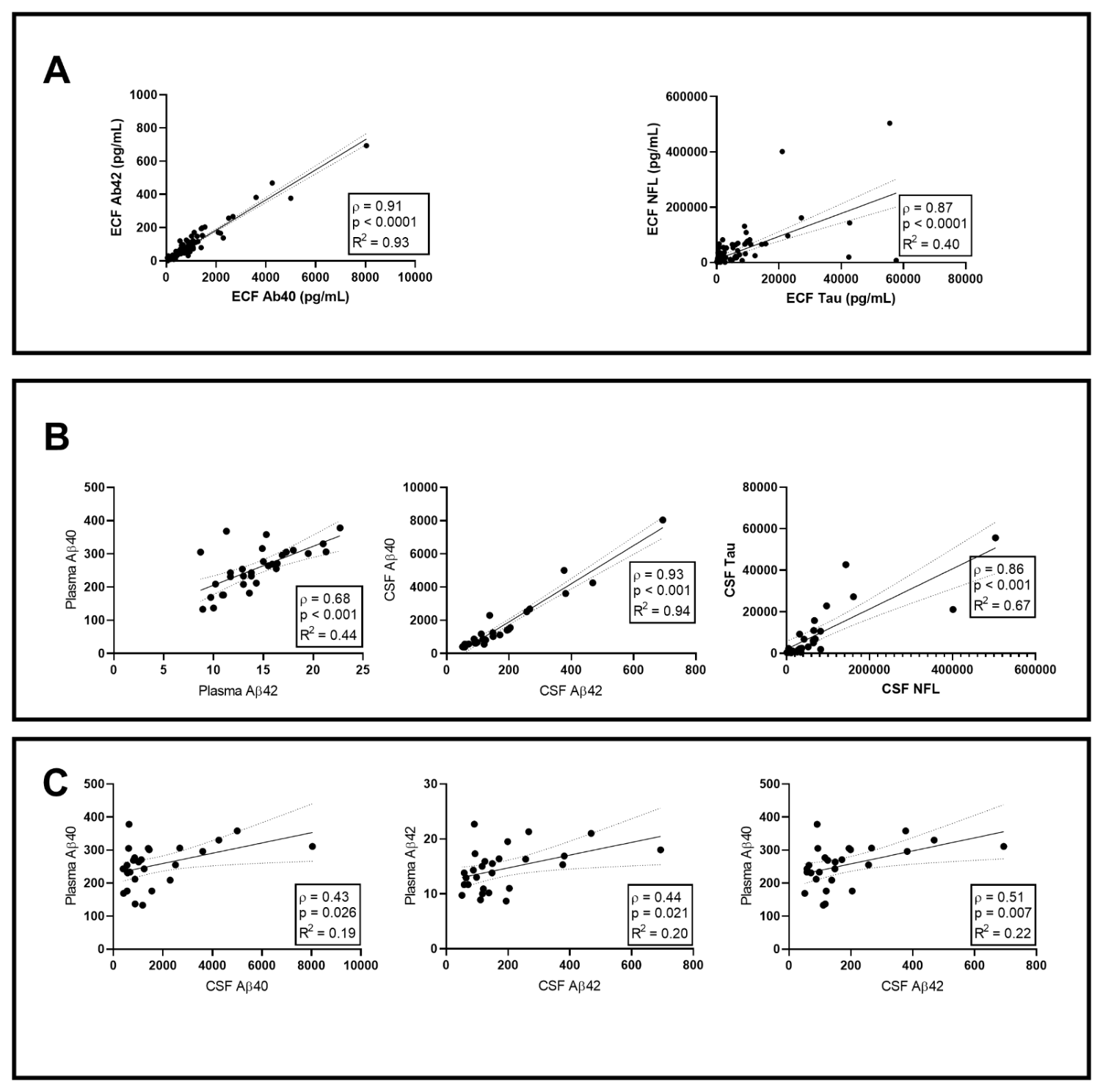


Supplementary Figure I: Linear regression of correlation between levels of biomarkers in three fluid compartments.

A) The levels of Aβ-40 and Aβ-42 in extracellular fluid (ECF) were highly correlated (ρ = 0.91, p< 0.0001) as were levels of neurofilament light (NF-L) and tau (ρ = 0.87, p < 0.0001). B) Plasma levels and cerebrospinal fluid (CSF) levels of Aβ-40 and Aβ-42 were highly correlated (ρ = 0.68 and 0.93, respectively, p < 0.001) as were CSF levels of tau and NF-L (ρ = 0.86, p < 0.001). C) Levels of Aβ-40 were moderately correlated in plasma and CSF, as were levels of Aβ-42 (ρ = 0.43 and 0.44 respectively, p < 0.05), and plasma levels of Aβ-40 with CSF levels of Aβ-42 (ρ0.51, p = 0.007).
